# Effect of Pumpkin Seed Protein Concentrate and Xanthan Gum on the Properties of Gluten-Free Frozen Batter and the Resulting Cake

**DOI:** 10.17113/ftb.64.02.26.9173

**Published:** 2026-06-15

**Authors:** Hoda Malekitabrizi, Mehran Aalami

**Affiliations:** 1 Master's Graduate in Food Technology, Gorgan University of Agricultural Sciences and Natural Resources, Mofatteh Boulevard, Gorgan, Iran; 2 Department of Food Science and Technology, Gorgan University of Agricultural Sciences and Natural Resources, Mofatteh Boulevard, Gorgan, Iran

**Keywords:** gluten-free, pumpkin seed protein concentrate, xanthan gum, frozen batter, cake quality, sensory evaluation

## Abstract

**Research background:**

The increasing demand for gluten-free products has necessitated improved formulations, particularly under frozen storage conditions. Although freezing and thawing often lead to quality deterioration, limited research has explored the role of additives in enhancing gluten-free frozen batter. This study investigates the effects of pumpkin seed protein concentrate (PSPC) and xanthan gum on the properties of gluten-free frozen batter and the resulting cake.

**Experimental approach:**

Batter samples were formulated with varying mass fractions of PSPC (0, 10 and 20 %) and xanthan gum (0, 0.1 and 0.2 %), then frozen at −18 °C for 30 and 60 days. After thawing, the physicochemical properties, including viscosity and density, of the batters were analysed. Ash, moisture, protein content, porosity, volume, crust colour, and sensory properties of the baked cakes were evaluated.

**Results and conclusions:**

Increasing the PSPC mass fraction significantly improved the viscosity and density of the batter, as well as the porosity and volume of the cakes (p<0.05). Higher PSPC mass fractions also led to a significant decrease in crust lightness and redness (p<0.05). Xanthan gum positively affected most quality attributes, although it significantly increased cake hardness (p<0.05). Sensory analysis showed that the formulation containing 10 % PSPC and 0.2 % xanthan gum, after 30 days of freezing, achieved the highest overall quality and consumer acceptance (p<0.05).

**Novelty and scientific contribution:**

The positive effect of PSPC and xanthan gum on the properties of gluten-free frozen batter and the resulting cakes was confirmed in the study. These findings contribute to the optimisation of gluten-free formulations that could be used to develop high-quality gluten-free frozen bakery products.

## INTRODUCTION

The use of frozen batter in bakery production has emerged as an innovative strategy to maintain product freshness after thawing. This approach offers several benefits, including increased consumer satisfaction, reduced labour and equipment costs, and extended shelf life (*1*, *2*). However, freezing and thawing processes present significant challenges to the final product quality, mainly due to the formation of large ice crystals that negatively affect texture and overall attributes (*3*).

To address these issues, precise temperature control and advanced storage techniques are essential to minimise quality degradation during freezing and distribution (*4*). Previous research has shown that storing batter at sub-zero temperatures significantly improves product quality compared to storage at higher temperatures. Furthermore, ingredient selection in batter formulations plays a crucial role in preserving the physicochemical and sensory properties of frozen baked goods. Additives such as hydrocolloids, emulsifiers, fats, and protein isolates can reduce freezing-induced damage, thereby enhancing texture and freshness (*5*–*8*). For example, the incorporation of gums into frozen batter helps regulate water migration during thawing, minimising structural damage (*9*). Hydrocolloids also delay staling, improving the quality of baked products made from frozen batter (*10*).

An important factor influencing frozen batter quality is the strength of the gluten network, with stronger networks typically yielding better texture and structure (*11*). However, the increasing prevalence of coeliac disease and gluten sensitivity has significantly increased demand for gluten-free bakery products. Currently, lifelong adherence to a gluten-free diet remains the only effective treatment for coeliac disease (*12*, *13*). Consequently, extensive research has focused on developing high-quality, nutritionally balanced gluten-free baked goods.

To improve the functional properties of gluten-free products, gluten alternatives such as hydrocolloids and protein isolates are essential (*14*, *15*). Hydrocolloids are hydrophilic, long-chain polymers that form gels in aqueous environments, stabilising batter and retaining gas during baking (*16*, *17*). Common hydrocolloids in gluten-free baking include hydroxypropyl methylcellulose (HPMC), carboxymethyl cellulose (CMC), and xanthan gum, all of which improve structure and volume in baked goods (*18*, *19*). Xanthan gum, a heteropolysaccharide composed of repeating pentasaccharide units, is particularly valued for its high solubility and viscosity, making it an effective thickening and stabilising agent (*20*).

Among gluten-free grains, rice is widely used due to its low sodium content, high digestibility, and nutritional value (*21*). In addition to serving as a carbohydrate source, rice provides essential vitamins and is cost-effective, making it an ideal base for gluten-free formulations. Pumpkin seeds (*Cucurbita pepo*), known for their rich content of bioactive compounds and essential nutrients, have also attracted attention for their health-promoting potential (*22*, *23*). These seeds contain 32–45 % protein and are rich in essential amino acids (*24*). The globulin fraction of pumpkin seed protein resembles that of legume proteins, highlighting its suitability as a functional and nutritious ingredient (*16*).

This study investigates the effects of pumpkin seed protein concentrate (PSPC), a protein-rich ingredient, and xanthan gum, a hydrocolloid, on the quality of gluten-free frozen batter and cakes. Although previous studies have examined the impact of freezing on batter and cake quality, no significant research has specifically addressed the role of additives such as PSPC and xanthan gum in gluten-free frozen batter. Therefore, the primary objective of this study is to address this research gap by evaluating the physicochemical and sensory effects of these additives in gluten-free cakes made from frozen batter. The control sample consists of frozen batter without PSPC or xanthan gum. Ultimately, this study aims to develop optimised, consumer-acceptable formulations for gluten-free frozen bakery products.

## MATERIALS AND METHODS

### Materials

Rice flour produced from the Tarom rice variety was purchased from a rice store in Gorgan, Golestan Province, Iran. The flour was sieved using a U.S. standard No. 80 sieve (aperture size approx. 180 µm) manufactured by Endecotts Ltd. (London, UK) to ensure uniform particle size. The sieved flour was then packed in triple-layered nylon bags and stored in a refrigerator at 4 °C. Fat-free pumpkin seed meal was obtained from Golestan Oil Factory (Gorgan, Golestan Province, Iran) and also packed in triple-layered nylon bags and refrigerated at 4 °C. Xanthan gum was supplied by Rhodia Food (Manchester, UK). Other ingredients, including sunflower oil (Rana Company, Tehran, Iran), powdered sugar (Golha Company, Mashhad, Iran), eggs (Telavang Company, Tehran, Iran), vanilla extract (Golha Company), dry milk powder (Pegah Golestan Dairy, Gorgan, Golestan Province, Iran), whey powder (Pegah Golestan Dairy), and baking powder (Golha Company), were sourced from reputable domestic producers. Purified water was obtained from a water purification system (WOSON® DRINK10, Jiangmen, PR China).

### Preparation of pumpkin seed protein concentrate

Pumpkin seed protein concentrate (PSPC) was prepared according to the method of Wang *et al*. (*25*), with minor modifications. Defatted pumpkin seed meal was mixed with 99.8 % ethanol at a 1:10 (*m*/*V*) ratio and incubated at 25 °C for 20 min with periodic shaking to enhance protein extraction, using a shaker incubator (JSSI-070; JS Research Inc., Gongju, South Korea). After incubation, the mixture was centrifuged at 10 000×*g* for 15 min using a high-speed centrifuge (Combi R515; Hanil, Incheon, South Korea). The supernatant was discarded, and the precipitate was placed under a fume hood to remove any residual ethanol by evaporation. Subsequently, the precipitate was dried in a hot-air oven at 25–30 °C for approx. 24 h, ground into a fine powder, and sieved through a U.S. standard No. 80 sieve (aperture size≈180 µm; Endecotts Ltd.) to obtain uniform particle size. The resulting concentrate was packed in polyethylene bags and stored at 4 °C until further use.

### Preparation of gluten-free frozen batter

Batter samples were prepared using the sugar-batter method described by Bennion and Bamford (*26*), with slight modifications. In this process, the amount of rice flour was varied, and PSPC was incorporated at three mass fractions (0, 10 and 20 %). Additionally, xanthan gum was added at mass fractions of 0, 0.1 or 0.2 % based on the total mass of the rice flour and PSPC mixture. Initially, sunflower oil (57 g) and powdered sugar (72 g) were mixed using a medium-speed mixer for 5 min until a light-coloured, creamy consistency was achieved. Eggs (72 g) were then gradually incorporated into the cream in three separate additions. The dry ingredients, *i.e.* flour (100 g), dry milk powder (2 g), whey powder (4 g), vanilla powder (0.5 g), and baking powder (2 g) were subsequently added to the mixture, and blending continued until a semi-smooth batter was obtained. Finally, water (30 g) was added, and mixing continued until a fully smooth and homogeneous batter was formed. The prepared batters were transferred into disposable containers, sealed tightly, and divided into two groups. The first group was stored in a freezer at −18 °C for 30 days, and the second group for 60 days.

### Preparation of gluten-free cake from frozen batter

After being frozen for 30 and 60 days, the batter samples were removed from the freezer and thawed in an incubator at 30 °C for 2 h. A mass of 50 g of each batter sample was then transferred into separate cake moulds and baked at 180 °C for 25 min in a conventional oven (UFP800TS; Memmert, Schwabach, Germany), following the method described by Bennion and Bamford (*26*). After baking, the cakes were removed from the oven and allowed to cool at room temperature for 1 h. They were then packaged in polyethylene bags (Vazin Plastic, Mahdasht, Alborz, Iran) and prepared for physicochemical and sensory evaluations. All tests were performed in triplicate.

### Chemical analysis

Chemical analyses were conducted on rice flour, fat-free pumpkin seed meal, PSPC, and gluten-free cake made from frozen batter. Moisture content was determined in triplicate by drying approx. 5 g of sample in a drying oven (UFP800TS; Memmert) at 105 °C for 4 h until constant mass was achieved, following AOAC Official Method 925.09 (*27*). Protein content was measured in triplicate using the Kjeldahl method with a digestion apparatus (model B150; Nabertherm, Lilienthal, Germany). Approximately 1 g of sample was digested with concentrated sulfuric acid and a catalyst, followed by distillation and titration of the released ammonia, according to AOAC Official Method 920.87 (*27*). Ash content was determined in triplicate by incinerating approx. 2 g of dried sample in a muffle furnace (model B150; Nabertherm) at 550 °C until white ash was obtained, indicating complete combustion, following AOAC Official Method 923.03 (*27*). All results are reported on a dry mass basis.

### Viscosity and density of batter after thawing

The viscosity of the batter sample was measured using a Brookfield rotational viscometer (model DV-II equipped with a Helipath stand; Brookfield Engineering Laboratories, Middleboro, MA, USA). According to the instrument manual and preliminary tests, the T-C spindle from the Helipath spindle set was selected. Viscosity measurements were recorded at a rotational speed of 20 rpm (*28*).

The density of batter samples was calculated by dividing the mass of a specific volume of batter by the mass of an equal volume of distilled water, following standard methods (*29*).

### Measurement of cake volume, porosity and hardness

The volume of cake samples was determined using the seed displacement method, following AACC Method 10-05.01 (*30*). First, the volume of an empty aluminium container was measured by filling it completely with millet seeds. After baking and cooling, each cake sample was removed, and the container was refilled with millet seeds to determine the displaced volume. The difference between the initial and refilled measurements was recorded as the volume of each cake sample (*30*).

The porosity of the gluten-free cake crumb was evaluated 2 h after baking using a digital image processing technique (*31*). A 2 cm×2 cm slice of the cake was scanned using an HP Scanjet G3010 scanner (Hewlett-Packard, Palo Alto, CA, USA) at a resolution of 600 dpi. The image was processed using ImageJ software (*32*). It was first converted to greyscale and then binarised. The resulting binary image consisted of light and dark pixels, with the ratio of light to dark pixels used as an index of porosity. A higher ratio indicated greater porosity in the cake texture. The porosity percentage was calculated using the Analyze function in ImageJ (*32*).

The hardness of the cake samples, measuring 2 cm×2 cm×2 cm, was evaluated 24 h after baking using a TA.XT Plus Texture Analyzer (Stable Microsystems, Surrey, UK). A 2 cm cubic piece of the cake, excluding the crust, was selected for testing. The P/36R probe (36 mm diameter) was used to compress the sample by 1 cm (50 % of its original height). Pre-test, test and post-test speeds were set at 2, 1 and 2 mm/s, respectively. The maximum force applied during compression, recorded in Newtons, represented the hardness of the sample. The testing procedure and parameters followed standard methods (*33*).

### Evaluation of cake crust colour

The colour of the cake crust was evaluated using the colour indices L*, a* and b*. The L* value indicates the lightness of the sample, ranging from 0 (black) to 100 (white). The a* value represents the colour range from red (+120) to green (−120), while the b* value covers the range from yellow (+120) to blue (−120). Two hours after baking, images of the crust were captured using an HP G3110 scanner (Hewlett-Packard, Palo Alto, CA, USA) at a resolution of 600 dpi (*34*). The images were then analysed using ImageJ software (*32*).

### Sensory evaluation of gluten-free cake made from frozen batter

The sensory evaluation of the cake samples was carried out by a panel of 15 semi-trained assessors (10 females and 5 males, aged 24 to 29). All assessors were graduate students at Gorgan University of Agricultural Sciences and Natural Resources who participated voluntarily in the study. The panel was considered ’semi-trained‘ because participants received brief training and instructions on the evaluation procedures, including the use of a 9-point hedonic scale, but they were not professionally trained sensory analysts. The evaluation took place in a controlled environment with standardised lighting, ambient room temperature, and absence of interfering odours or noise. Each sample was coded with a random three-digit number and presented in a randomised order to minimise bias. Water was provided for palate cleansing between samples. Assessors rated sensory attributes including appearance, aroma, texture, taste and overall acceptability. The test followed general guidelines for hedonic testing as described by Lawless and Heymann (*35*).

### Statistical analysis

All tests were conducted in triplicate. Data were analysed using a completely randomised factorial design with generalised linear models (GLM) in SAS software v. 9.1.3 (*36*). Significant differences among treatments were determined by one-way analysis of variance (ANOVA), and mean values were compared using Duncan’s multiple range test at a 95 % confidence level (*36*).

## RESULTS AND DISCUSSION

### Chemical composition of rice flour, fat-free pumpkin seed meal and protein concentrate

The chemical composition of rice flour, fat-free pumpkin seed meal and pumpkin seed protein concentrate (PSPC), including moisture, ash and protein content, is summarised in Table 1. The fat-free pumpkin seed meal initially contained 48.57 % protein and 3.35 % ash. After the protein enrichment process applied to the pumpkin seed material, the protein mass fraction increased to 59.78 %, while the ash mass fraction increased to 7.05 %. The increase in ash mass fraction may be attributed to a concentration effect resulting from the removal of lipids and carbohydrates during processing, leading to a relative increase in the amount of mineral components. The relatively high protein mass fraction of PSPC makes it a promising ingredient for enhancing the nutritional profile of gluten-free formulations.

**Table 1:** Chemical composition of rice flour, pumpkin seed meal and pumpkin seed protein concentrate (PSPC) on dry mass basis

	*w*/%		
Component	Moisture	Ash	Protein
Rice flour	9.29±0.07	0.63±0.02	8.20±0.1
Fat-free pumpkin seed meal	(7.35±0.06)^a^	(3.4±0.1)^b^	(48.6±0.5)^b^
PSPC	(6.24±0.5)^b^	(7.0±0.2)^a^	(59.8±0.2)^a^

Results are expressed as mean value±standard deviation (S.D.). Different letters in superscript within the same column indicate statistically significant differences (p≤0.05) according to Duncan’s multiple range test. Note: Rice flour was not included in the statistical analysis due to its compositional differences compared to the pumpkin-based samples

### Moisture content of cake made from frozen batter

**Fig. 1:**
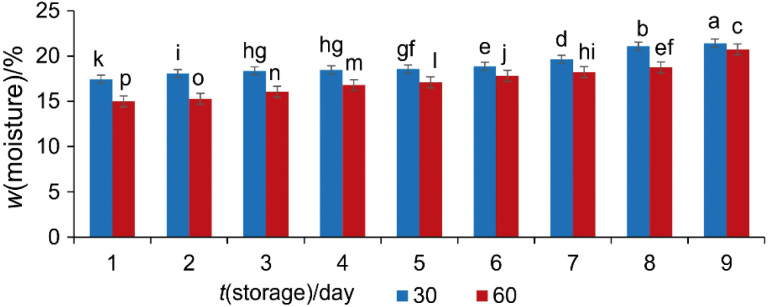
Effect of pumpkin seed protein concentrate (PSPC) and xanthan gum on the moisture content of cake samples made from frozen batter after 30 and 60 days of storage. Treatments numbered on the x-axis correspond to the following formulations: 1=control; 2 and 3=0 % PSPC and 0.1 and 0.2 % xanthan gum, respectively; 4, 5 and 6=10 % PSPC and 0, 0.1 and 0.2 % xanthan gum, respectively; 7, 8 and 9=20 % PSPC and 0, 0.1 and 0.2 % xanthan gum. Values with different letters indicate significant differences according to Duncan’s test (p≤0.05)

The results (Fig. 1) showed that the addition of PSPC and xanthan gum significantly (p<0.05) improved moisture retention in the cakes. These additives led to higher moisture mass fraction than in the control samples. Moreover, the duration of freezing negatively affected the moisture quality of the cakes, as those frozen for 30 days retained higher moisture mass fraction than those frozen for 60 days. Among the experimental samples, the highest moisture mass fraction was found in the sample containing 20 % PSPC and 0.2 % xanthan gum. Xanthan gum is widely recognised for its high water-holding capacity, which prevents the formation of large ice crystals during freezing and reduces water migration during thawing (*37*). Previous studies have shown that xanthan gum significantly aids moisture retention during both baking and post-baking stages, thereby extending the shelf life of frozen bakery products (*38*). Additionally, the incorporation of PSPC, which is rich in proteins and natural fibre, enhances the water-holding capacity and moisture retention in cakes (*39*).

### Ash and protein content of cake made from frozen batter

**Fig. 2:**
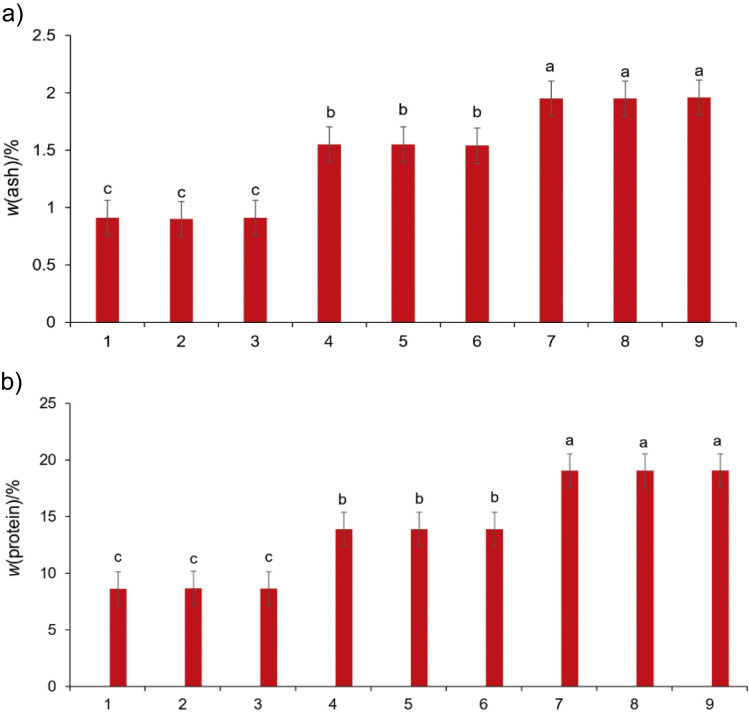
Effect of pumpkin seed protein concentrate (PSPC) and xanthan gum on: a) ash content, and b) protein content of cake samples made from frozen batter. Treatments numbered on the x-axis correspond to the following formulations: 1=control; 2 and 3=0 % PSPC and 0.1 and 0.2 % xanthan gum, respectively; 4, 5 and 6=10 % PSPC and 0, 0.1 and 0.2 % xanthan gum, respectively; 7, 8 and 9=20 % PSPC and 0, 0.1 and 0.2 % xanthan gum. Values with different letters indicate significant differences according to Duncan’s test (p≤0.05)

The ash content of the cake samples was significantly affected by the incorporation of PSPC. Specifically, increasing the mass fraction of PSPC in the formulations led to a statistically significant increase in ash mass fraction (p<0.05). This increase can be attributed to the higher mineral content of pumpkin seed flour than of rice flour. As shown previously (Table 1), the ash mass fraction of PSPC is substantially higher than that of rice flour, which explains the observed increase in overall ash mass fraction with higher PSPC substitution. Additionally, Fig. 2a clearly illustrates the upward trend in ash mass fraction corresponding to increasing mass fractions of PSPC in the cake formulations. In contrast, the addition of xanthan gum had no significant effect on the ash content (p>0.05), indicating that it does not contribute meaningfully to the mineral composition of the final product. Furthermore, no statistically significant differences were observed in ash mass fraction between samples prepared from dough stored for 30 days and those prepared from dough stored for 60 days. These findings suggest that the incorporation of pumpkin seed flour can effectively enhance the mineral content and overall nutritional profile of gluten-free baked products (*40*).

According to the results shown in Fig. 2, cakes formulated with higher mass fractions of PSPC showed a significant increase in protein content compared to the control samples (p<0.05). This increase can be primarily attributed to the higher protein mass fraction of PSPC (59.78 %) than of rice flour (8.20 %). Replacing rice flour with PSPC effectively enhanced the protein mass fraction of the cakes, which is particularly important for improving the nutritional profile of gluten-free products. These findings are consistent with previous studies by Voučko *et al.* (*41*) and Tomić *et al.* (*40*). On the other hand, the addition of xanthan gum did not significantly affect the protein content in this study. Additionally, the duration of dough storage under frozen conditions did not have a significant effect on the protein content of the cakes. This lack of effect may be attributed to the characteristics of the Kjeldahl method, which measures protein content based on total acids during storage. Therefore, the absence of changes in protein mass fraction after frozen dough storage could be related to the limitations of this method in detecting such structural alterations.

### Viscosity and density changes in batter after thawing

**Fig. 3:**
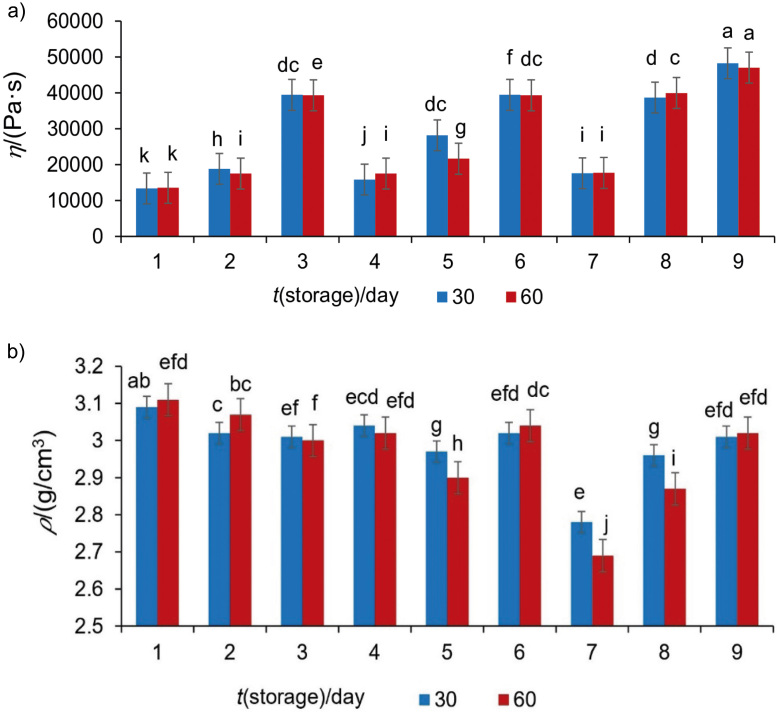
Effect of pumpkin seed protein concentrate (PSPC) and xanthan gum on: a) viscosity, and b) density of frozen batter samples after 30 and 60 days of storage. Treatments numbered on the x-axis correspond to the following formulations: 1=control; 2 and 3=0 % PSPC and 0.1 and 0.2 % xanthan gum, respectively; 4, 5 and 6=10 % PSPC and 0, 0.1 and 0.2 % xanthan gum, respectively; 7, 8 and 9=20 % PSPC and 0, 0.1 and 0.2 % xanthan gum. Values with different letters indicate significant differences according to Duncan’s test (p≤0.05)

According to the results presented in Fig. 3a, the addition of xanthan gum significantly (p<0.05) increased the viscosity of the cake batter. Similarly, PSPC also improved viscosity, and the synergistic effect of these two ingredients further enhanced this property. However, no significant difference in the viscosity of the frozen batters was observed between samples frozen for 30 and 60 days. Freezing and thawing processes generally reduce batter quality due to the physical stresses imposed on the matrix, which disrupt its ability to retain moisture. This leads to an increase in free water and, consequently, a reduction in viscosity (*28*, *42*). However, the addition of hydrocolloids such as xanthan gum significantly increases batter viscosity and prevents its reduction under freezing conditions (*43*). This effect is attributed to the high water-binding capacity of hydrocolloids, which compete with the batter polymers (such as starch and proteins) to reduce water activity and prevent moisture migration (*16*). PSPC, with its high protein content and water-holding capacity, combined with xanthan gum, which dissolves in cold water and forms gel-like networks, effectively increased batter viscosity (*20*).

Batter density plays a crucial role in air bubble retention, which directly influences the final cake volume (*44*). Lower batter density is generally associated with better air retention and, consequently, a higher final product volume. In this study, the effects of different mass fractions of PSPC and xanthan gum on batter density were evaluated after 30 and 60 days of frozen storage. As shown in Fig. 3b, the addition of PSPC up to 10 % in combination with xanthan gum up to 0.2 % significantly reduced batter density (p<0.05). This reduction was more pronounced after 60 days of storage than after 30 days. Specifically, the combination of 10 % PSPC and 0.1 % xanthan gum resulted in the lowest batter density after 60 days, indicating a stronger batter matrix, optimal viscosity, and improved air bubble retention. In contrast, increasing the PSPC mass fraction to 20 %, particularly when combined with 0.2 % xanthan gum, led to an increase in batter density. This increase is likely due to excessive viscosity, which hindered proper air incorporation during mixing, thereby reducing aeration and increasing density. The sample containing 20 % PSPC and 0.2 % xanthan gum showed one of the highest densities across both storage durations. Furthermore, comparison of the two storage periods revealed that batter density generally increased after 60 days. This trend may be attributed to microstructural changes in the batter caused by ice crystal formation and recrystallisation during prolonged frozen storage, which could negatively affect air bubble stability. These findings are consistent with previous studies reporting that low mass fractions of hydrocolloids such as xanthan gum tend to reduce batter density, while higher mass fractions may have the opposite effect due to increased viscosity that interferes with air incorporation (*28*, *45*).

### Changes in volume, porosity and hardness of cake made from frozen batter

**Fig. 4:**
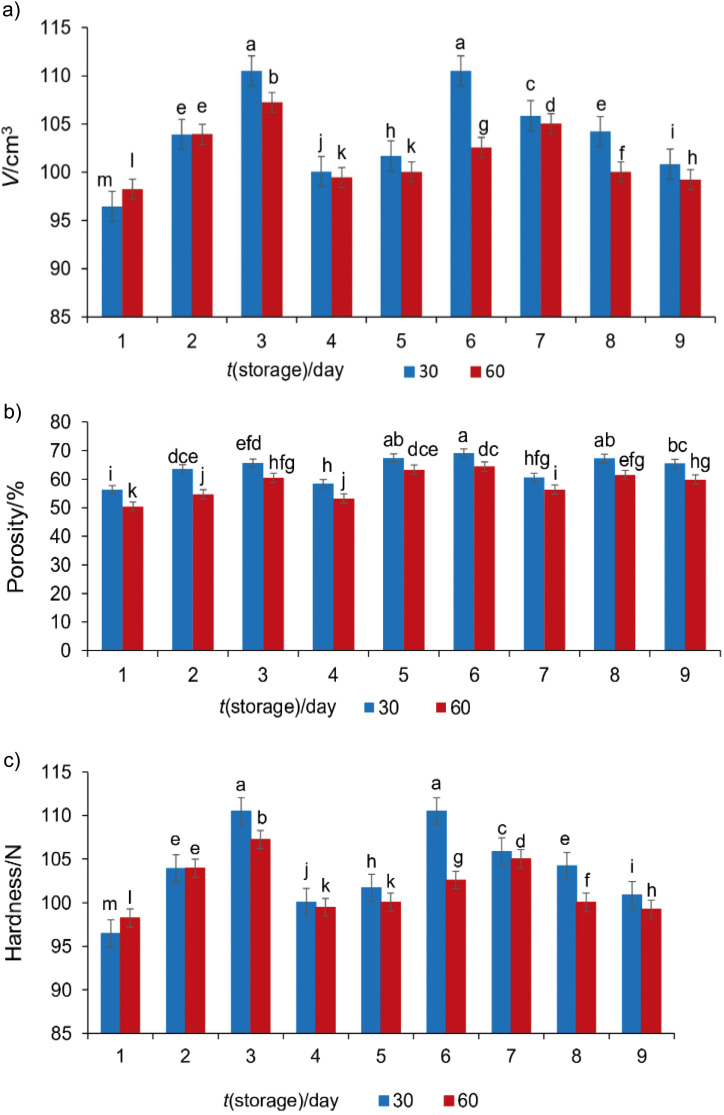
Effect of pumpkin seed protein concentrate (PSPC) and xanthan gum on: a) volume, b) porosity, and c) hardness of cake samples made from frozen batter after 30 and 60 days of storage. Treatments numbered on the x-axis correspond to the following formulations: 1=control; 2 and 3=0 % PSPC and 0.1 and 0.2 % xanthan gum, respectively; 4, 5 and 6=10 % PSPC and 0, 0.1 and 0.2 % xanthan gum, respectively; 7, 8 and 9=20 % PSPC and 0, 0.1 and 0.2 % xanthan gum. Values with different letters indicate significant differences according to Duncan’s test (p≤0.05)

Cake volume is a critical quality attribute that directly influences consumer acceptance of baked products (*28*). It reflects not only the visual appeal of the final product but also the ability of the batter to retain gas during baking, indicating the effectiveness of leavening and structural development processes. As shown in Fig. 4a, the incorporation of PSPC and xanthan gum generally resulted in a significant increase in cake volume compared to the control sample (p<0.05). The highest volume was observed in the formulation containing 10 % PSPC and 0.2 % xanthan gum (p<0.05), suggesting a synergistic interaction between these ingredients in enhancing batter structure and gas retention. However, this trend was not consistent across all formulations. Notably, the sample containing 20 % PSPC alone had a greater volume than the sample with 10 % PSPC and 0.1 % xanthan gum (p<0.05), indicating that, in some cases, a higher PSPC mass fraction alone may be more beneficial than its combination with a low mass fraction of xanthan gum. In contrast, the formulation containing 20 % PSPC and 0.2 % xanthan gum showed the lowest volume among all enriched samples (p<0.05). This reduction in volume appears to be multifactorial. Excessive batter viscosity likely hinders air incorporation during mixing and destabilises air bubbles during freezing, thus limiting volume expansion during baking (*28*). Furthermore, at high mass fractions, both PSPC and xanthan gum may compete with rice starch for available water, impairing starch gelatinisation and weakening the structural integrity of the cake. Additionally, the high protein mass fraction in PSPC can promote protein aggregation, forming a rigid network that restricts gas cell expansion during baking. Overall, moderate increases in batter viscosity due to PSPC and xanthan gum can support air bubble stability and enhance cake volume. However, when viscosity becomes excessively high, especially at increased mass fractions of these additives, it may lead to insufficient aeration and compromised starch functionality, ultimately reducing final cake volume. In addition to formulation, frozen storage duration had a significant effect on cake volume. Cakes produced from batter stored at −18 °C for 30 days consistently had higher volume than those stored for 60 days (p<0.05). The observed reduction in volume after 60 days of frozen storage is likely attributed to gradual degradation of the batter matrix due to ice crystal formation and recrystallisation, which reduces the ability of the batter to retain air post-thawing and restricts expansion during baking (*28*).

The results of porosity analysis, as shown in Fig. 4b, show that increasing the PSPC mass fraction significantly increases cake porosity compared to the control sample (p<0.05). Similarly, the addition of xanthan gum improves porosity, corroborating previous findings that xanthan gum facilitates better gas distribution within the batter matrix (*44*). The synergistic effect of PSPC and xanthan gum is particularly evident in the sample containing 10 % PSPC and 0.2 % xanthan gum, which shows the highest porosity after 30 days of frozen storage. This suggests that the combination of PSPC and xanthan gum contributes to a more stable batter structure, enhancing effective gas cell entrapment during baking. However, although the overall trend indicates increased porosity with the addition of PSPC and xanthan gum, this effect is not consistently maintained over time. Notably, samples stored for 60 days show a decline in porosity, likely due to ice crystal formation and recrystallisation, which can damage gas cells and compromise the structural integrity of the cake matrix. This highlights the impact of prolonged frozen storage on the physical properties of batter systems. These findings are consistent with previous studies reporting the positive influence of pumpkin seed flour on porosity (*38*), as well as the functional benefits of hydrocolloids like xanthan gum in maintaining aeration through improved gas retention and viscosity modulation (*44*).

Cakes produced from frozen batter, 24 h after baking exhibited a significant reduction in hardness with the addition of PSPC (Fig. 4c), while the inclusion of xanthan gum significantly increased cake hardness (p<0.05). The reduction in hardness observed in samples containing pumpkin seed protein concentrate was attributed to the formation of larger pores within the cake structure. However, Shi *et al*. (*45*) reported that the addition of protein peptides to frozen batter resulted in reduced cake hardness after freezing and improved texture. Storage time also had a significant effect on cake hardness (*46*). Prolonged storage, particularly in samples stored for 60 days, led to increased hardness. On the other hand, the addition of xanthan gumsignificantly increased the number of pores in the cakes, contributing to the formation of smaller, more uniform holes. This prevented excessive air expansion and helped maintain the cake structure under the forces applied during texture analysis. These results indicate that the formulation of frozen batter, particularly with the addition of protein isolates and hydrocolloids, significantly affects the structural and textural properties of baked products during frozen storage.

### Changes in crust colour of cake made from frozen batter

**Fig. 5:**
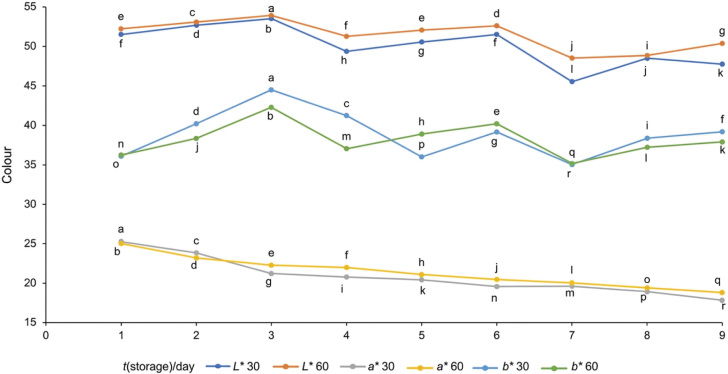
Effect of pumpkin seed protein concentrate (PSPC) and xanthan gum on the colour (L, a*, b*) of cake samples made from frozen batter after 30 and 60 days of storage. Treatments numbered on the x-axis correspond to the following formulations: 1=control; 2 and 3=0 % PSPC and 0.1 and 0.2 % xanthan gum, respectively; 4, 5 and 6=10 % PSPC and 0, 0.1 and 0.2 % xanthan gum, respectively; 7, 8 and 9=20 % PSPC and 0, 0.1 and 0.2 % xanthan gum. Values with different letters indicate significant differences according to Duncan’s test (p≤0.05)

The colour characteristics of the crust of gluten-free cakes produced from batter stored under freezing conditions for 30 and 60 days were investigated. The results in Fig. 5 show that the addition of PSPC significantly decreased the lightness (L*) of the samples compared to the control (p<0.05). This reduction in lightness is likely due to increased Maillard reaction intensity during baking. However, the addition of xanthan gum to the formulation reduced the darkening effect caused by PSPC and significantly increased the lightness of the samples (p<0.05). This effect is mainly attributed to the role of hydrocolloids in promoting more uniform moisture distribution and reducing the intensity of Maillard browning reactions (*47*). Since pumpkin seeds contain high amounts of sulfur-containing amino acids, the darkening effect observed with PSPC addition is likely related to reactions between free amino acids and reducing sugars during baking. Regarding colour indices, the a* (redness) value indicated that the addition of PSPC and xanthan gum significantly reduced the redness of the samples compared to the control (p<0.05). Furthermore, the b* (yellowness) value significantly increased in samples containing PSPC and xanthan gum compared to the control (p<0.05). These results agree with previous findings reporting significant changes in the colour and texture of gluten-free bread when plant flour such as pumpkin seed flour were added (*40*, *48*). Additionally, freezing duration had a significant effect on the darkening of the cake crust. Longer freezing time, especially 60 days, led to darker final products. These findings suggest that both freezing duration and the additives used can considerably affect the colour characteristics and overall quality of gluten-free cakes.

### Evaluation of sensory properties of cake made from frozen batter

**Fig. 6:**
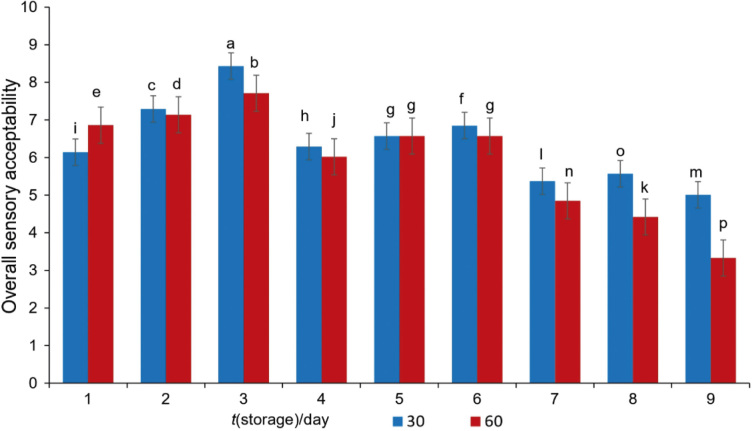
Effect of pumpkin seed protein concentrate (PSPC) and xanthan gum on overall sensory acceptability of cake samples made from frozen batter after 30 and 60 days of storage. Treatments numbered on the x-axis correspond to the following formulations: 1=control; 2 and 3=0 % PSPC and 0.1 and 0.2 % xanthan gum, respectively; 4, 5 and 6=10 % PSPC and 0, 0.1 and 0.2 % xanthan gum, respectively; 7, 8 and 9=20 % PSPC and 0, 0.1 and 0.2 % xanthan gum. Values with different letters indicate significant differences according to Duncan’s test (p≤0.05)

The sensory evaluation of cakes made from frozen batter stored for 30 to 60 days showed that the addition of xanthan gum significantly improved sensory attributes, including colour, appearance, aroma, taste and texture (p<0.05). Conversely, increasing the mass fraction of PSPC resulted in lower sensory scores across all evaluated attributes. Based on these results, the sample containing 0.2 % xanthan gum without PSPC, from batter stored for 30 days, was identified as having the highest sensory acceptance. Among the PSPC-containing samples, the formulation with 10 % PSPC and 0.2 % xanthan gum received the highest sensory scores and was considered the optimal substitution amount. Additionally, storage duration of the frozen batter significantly affected sensory attributes, with cakes stored for 30 days showing markedly superior sensory characteristics. Due to the large number of treatments (18) and multiple sensory attributes evaluated, only the overall acceptability scores are presented for clarity and better visualisation. The results shown in Fig. 6 indicate that the addition of xanthan gum to the frozen batter formulation, particularly after 30 days, resulted in higher sensory scores for both PSPC and xanthan gum than other formulations with different mass fractions of PSPC and xanthan gum. A recent study by Galenko *et al.* (*49*) also investigated the effect of pumpkin seed protein concentrate in cakes. Although that study did not focus on frozen batter, it found that adding 10 % PSPC led to higher sensory acceptance for taste, texture and overall appeal. To the best of our knowledge, this study is the first to investigate the use of PSPC in cakes made from frozen batter. The findings clearly emphasise that careful selection of the optimal substitution mass fractions and ingredient combination significantly affects the sensory attributes and overall quality of baked products.

## CONCLUSIONS

This study demonstrates the potential of pumpkin seed protein concentrate (PSPC) and xanthan gum as effective functional ingredients in gluten-free cake formulations intended for frozen storage. By partially replacing rice flour with PSPC and adding xanthan gum, the nutritional value, structural integrity, and sensory properties of the cakes were improved. The optimal formulation, containing 10 % PSPC and 0.2 % xanthan gum provided a balanced improvement in appearance, texture and consumer acceptance after 30 days of frozen storage. The findings highlight the influence of freezing and storage on the performance of hydrocolloids and protein concentrates in batter systems, emphasising the need to consider these factors in product development. These results offer new insights into optimising gluten-free products for long-term frozen storage, providing practical solutions for both health-conscious consumers and the food industry.
